# Bactericidal Effect of Low Temperature Plasma Combined with Slightly Acidic Electrolyzed Water Against *Listeria monocytogenes*

**DOI:** 10.3390/foods15091458

**Published:** 2026-04-22

**Authors:** Jiayi Shi, Zhanfei Wang, Bing Li, Xingzhe Zhang, Zhanpeng Wu, Jianxiong Hao, Tongjiao Wu

**Affiliations:** 1 College of Food and Biology, Hebei University of Science and Technology, Shijiazhuang 050018, China; 15613900362@163.com (J.S.); 19080634869@163.com (Z.W.); li2003lib@163.com (B.L.); z15720098196@163.com (X.Z.); 2 Shijiazhuang Huikang Food Co., Ltd., Shijiazhuang 050802, China; wuzhanpeng@chinahuikang.com

**Keywords:** low temperature plasma (LTP), slightly acidic electrolyzed water (SAEW), *Listeria monocytogenes*, bactericidal effect, combined treatment

## Abstract

This study investigated the bactericidal effect and examined the associated cellular damage of low temperature plasma (LTP) combined with slightly acidic electrolyzed water (SAEW) against *Listeria monocytogenes*. Single-factor experiments were conducted to assess the bactericidal efficacy under individual treatment conditions, followed by the evaluation of three different combination sequences. An orthogonal experimental design was performed to optimize the key parameters, and the optimal treatment conditions were determined as LTP at 45 W with an electrode spacing of 1 mm for 2 min, combined with SAEW at an available chlorine concentration (ACC) of 30 mg/L. Under these conditions, confocal laser scanning microscopy (CLSM) with SYTO 9/PI staining confirmed that the combined treatment caused cell death, as indicated by loss of membrane integrity in treated cells. A resuscitation assay further ruled out the viable but non-culturable (VBNC) state, as no bacterial growth was detected after 48 h of enrichment. The leakage of intracellular proteins and nucleic acids was measured using the Coomassie Brilliant Blue method combined with a microplate reader, and changes in cellular morphology were observed by scanning electron microscopy (SEM). The results demonstrated that SAEW+LTP treatment exerted a distinct effect, significantly disrupting bacterial cell membrane integrity, inducing the leakage of intracellular contents, and causing obvious morphological damage to the bacterial cells. In conclusion, the combined treatment of LTP and SAEW significantly improved the bactericidal efficiency against *L. monocytogenes*, which may be due to the combined disruptive effects on membrane integrity and subsequent structural and functional damage to the cells. Future investigations are needed to unravel the precise mechanisms, establish the efficacy against a wider panel of strains, and explore the potential for practical application in food matrices.

## 1. Introduction

Foodborne illnesses have become a significant concern in global public health. Caused by pathogenic microorganisms in food, these diseases pose a dual risk to human well-being and the reliability of the food supply chain. In China, contamination with foodborne pathogens remains a primary threat to food safety. In 2022, microbial pathogens were responsible for the highest number of reported foodborne disease cases nationwide, accounting for 38.43% [1]. Therefore, research on the prevention and control of foodborne pathogens has become one of the core topics in the field of food safety [2].

*Listeria monocytogenes* is a highly representative pathogenic microorganism. As a Gram-positive, facultatively anaerobic coccobacillus, it exhibits exceptional environmental adaptability, surviving for up to one year even under storage conditions at −20°C. *L. monocytogenes* is prevalent in dairy, meat, and seafood products [2,3]. After invading the organism through contaminated food, it can attack the nervous and circulatory system, causing diseases such as diarrhea, fever, meningitis, and sepsis, with a mortality rate as high as 30–35%. It poses particularly significant hazards to immunocompromised populations, including pregnant women, newborns, patients with chronic diseases, and the elderly. Pregnant women infected with *L. monocytogenes* may lead to fetal arrest or miscarriage [4].

Thermal and chemical sterilization are currently the predominant technologies in the food industry, they are associated with major drawbacks. Although thermal sterilization technologies (such as high temperature cooking and pasteurization) could effectively eliminate microorganisms, the high temperatures required during processing would destroy nutrients such as vitamins and proteins in food. They also alter the original flavor of food, thereby reducing food quality [5,6]. Chemical sterilization technologies rely on preservatives, disinfectants and other chemical agents to inhibit or kill microorganisms. However, these agents may remain as residues in food. Long-term consumption of food containing residual chemical poses potential health risks to humans [7,8]. Furthermore, certain chemical agents may also cause environmental pollution, which contradicts the principles of sustainable development. Individual non-thermal sterilization technologies, such as ultrasound, high-voltage pulsed electric fields, and ultraviolet irradiation, can effectively preserve food quality [9,10,11], their bactericidal efficacy is inherently limited [12]. In contrast, the combination of multiple technologies has been demonstrated to significantly enhance bactericidal efficacy while causing less impact on food quality [13,14,15]. Consequently, the exploration of combined multi-technology approaches has emerged as a research hotspot.

Among various non-thermal technologies, low-temperature plasma (LTP) and slightly acidic electrolyzed water (SAEW) have attracted considerable attention due to their unique bactericidal mechanisms [16,17,18]. As the fourth state of matter, LTP is composed of a large number of charged particles and neutral particles, which can be generated through gas discharge methods such as corona discharge and dielectric barrier discharge. The high-energy particles, active substances (such as free radicals), and ultraviolet radiation contained in LTP can destroy the cellular structure of microorganisms (including breaking down cell membranes and causing the leakage of intracellular contents), oxidize DNA molecules (resulting in DNA damage and inhibiting microbial reproduction), thereby achieving efficient sterilization. Moreover, LTP has little impact on the nutrients and flavor of food and does not introduce harmful substances during the treatment process [19,20]. SAEW, primarily composed of hypochlorous acid with a pH range of 5.0–6.5, exhibits short sterilization time and broad-spectrum efficacy. It has been recognized as a food additive-grade sterilizing component, which is non-toxic by oral administration, can effectively eliminate various microorganisms without leaving harmful residues, and also has advantages such as water conservation and safe use [21,22].

The complementary nature of the action targets between LTP and SAEW is evident, LTP primarily disrupts cell membranes and DNA, whereas SAEW mainly oxidizes intracellular proteins via hypochlorous acid, attacking bacterial cells through distinct pathways, the combination of these two technologies is expected to achieve enhanced bactericidal efficacy [23,24]. In contrast, other commonly used combined sterilization strategies, such as ultrasound combined with ultraviolet irradiation, often target similar cellular components [25], resulting in limited synergistic effects. Nevertheless, to date, only limited studies have been reported on the application of LTP combined with SAEW for the inactivation of foodborne pathogens.

In this study, LTP and SAEW were combined for the first time to systematically evaluate their bactericidal efficacy against *Listeria monocytogenes* and to preliminarily elucidate the underlying mechanisms, aiming to provide a theoretical basis for the development of novel, efficient, and green sterilization technologies, and to lay a foundation for their future application in real food matrices.

## 2. Materials and Methods

### 2.1. Activation of Strains and Preparation of Bacterial Suspension

*Listeria monocytogenes* (ATCC 19114) used in the experiment was purchased from Beijing Solarbio Biotechnology Co., Ltd., Beijing, China. This strain was isolated from the brain of a sheep with circling disease and belongs to serotype 4a, which is commonly found in animal listeriosis and natural environments. This strain serves as a well-established reference in food microbiology studies, possessing a clearly defined genetic background that supports its use in the preliminary exploration of the underlying mechanisms.

The *L. monocytogenes* strain was streaked and inoculated onto PALCAM agar (Beijing Land Bridge Technology Co., Ltd., Beijing, China) plates using sterile tweezers to pick up porcelain beads containing the bacteria. The plates were then incubated at 37 °C for 24 h. Typical single colonies were inoculated into 5 mL of TSB-YE liquid medium and shake-cultured at 37 °C and 160 rpm for 24 h. Aliquots (0.1 mL) of the bacterial culture were plated onto PALCAM agar and incubated at 37 °C for 24 h to obtain purified secondary colonies. To prepare the initial bacterial suspension, 9 mL of normal saline was added to the plate, and the colonies were scraped off. The suspension was dispensed into three centrifuge tubes (3 mL per tube) and centrifuged at 3000× *g* for 10 min. The supernatant was removed, and the pellets were resuspended in 1 mL of normal saline per tube, followed by vortex mixing. After pooling the suspensions, two additional rounds of centrifugation and resuspension were performed to remove residual medium components. Finally, the pellet was taken up in 1 mL of normal saline. The OD600 of the bacterial suspension was measured using a spectrophotometer and adjusted to 1.0 ± 0.01 to obtain the bacterial suspension for subsequent experiments [26].

### 2.2. Single-Factor Bactericidal Experiment of Low Temperature Plasma

#### 2.2.1. Low Temperature Plasma Equipment

The low temperature plasma equipment (LTP-2000K) used in the experiment was purchased from Opps Plasma Technology Co., Ltd., Suzhou, Jiangsu, China. The device generates plasma via dielectric barrier discharge (DBD), with stainless steel electrodes and ambient air as the working gas. The electrode spacing was adjustable, and the treatment time and power were set according to the experimental requirements.

#### 2.2.2. Bactericidal Efficacy of Low Temperature Plasma with Different Power

The equipment power gradient was set as 40 W, 45 W, 50 W, 55 W, and 60 W, with the electrode spacing (1 mm) and treatment time (5 min) fixed as constant parameters to treat *L. monocytogenes*. After treatment, 100 μL of the bacterial suspension was plated onto TSB-YE agar containing 0.6% yeast extract and then incubated at 37 °C for 24 h. Colonies were counted, and the bactericidal efficacy under each power condition was calculated. When no colonies were observed, the detection limit was set at 1.0 log CFU/mL (based on a plating volume of 100 μL).

#### 2.2.3. Bactericidal Efficacy of Low Temperature Plasma with Different Treatment Time

The treatment time gradient was set at 1 min, 3 min, 5 min, and 7 min, with the power (50 W) and electrode spacing (1 mm) fixed as constant parameters. The target bacteria were treated under these conditions. Subsequently, the treated bacterial solutions were plated onto agar plates to observe the bactericidal efficacy of the different treatment times.

#### 2.2.4. Bactericidal Efficacy of Low Temperature Plasma with Different Electrode Spacing

The gradient of electrode spacing was set as 1 mm, 3 mm, 5 mm, 7 mm, with the power (50 W) and treatment time (5 min) fixed as constant parameters to treat the target bacteria. The treated bacterial solutions were plated onto agar plates to observe the bactericidal efficacy of different electrode spacing.

### 2.3. Single-Factor Bactericidal Experiment of Slightly Acidic Electrolyzed Water

#### 2.3.1. Preparation of Slightly Acidic Electrolyzed Water and Determination of Physical and Chemical Indicators

Prepare SAEW with reference to the method of He et al. [27]. adjust pH value to 5.85–5.90, and determine its available chlorine concentration (ACC) by iodometry [28]. ACC calculation formula is shown in Equation.

(1) ACC = (c × V_1_ × 35.45 × D) ÷ V_2_

where c (mol/L), V_1_ (L), V_2_ (L), and D represent the concentration of Na_2_S_2_O_3_ standard solution, the titrant volume consumed, the sample volume, and the dilution factor, respectively.

#### 2.3.2. Bactericidal Efficacy of Slightly Acidic Electrolyzed Water with Different Available Chlorine Concentrations

The SAEW preparation method described by Li et al. [29] was used with minor modifications. SAEW solutions with varying ACC (10, 20, 30, 40, and 50 mg/L) were prepared. 1 mL of suspension was added to 9 mL of electrolyzed water with different ACC, treated for 5 min at room temperature, and then 1 mL of 1 mol/L sodium thiosulfate was added to terminate the reaction. After treatment, the bacterial suspensions were plated onto agar plates to evaluate the bactericidal efficacy at different ACC levels.

#### 2.3.3. Bactericidal Efficacy of Slightly Acidic Electrolyzed Water with Different Treatment Times

A 1 mL aliquot of the bacterial suspension was mixed with 9 mL of SAEW containing 30 mg/L ACC and incubated at room temperature for 1, 3, 5, or 7 min, and then sodium thiosulfate was added to terminate the reaction. The treated bacterial suspensions were then plated onto agar plates to evaluate the bactericidal efficacy at different treatment times.

### 2.4. Determination of Optimum Treatment Sequence of Low Temperature Plasma-Slightly Acidic Electrolyzed Water Combined Treatment

#### 2.4.1. Bactericidal Efficacy of the Sequential Treatment of LTP Followed by SAEW Treatment

According to the single-factor experimental results, the bacterial suspension underwent initial treatment using selected LTP parameters, including power, treatment time, and electrode spacing. Subsequently, the treated suspension was transferred into SAEW for secondary treatment according to predefined parameters, and the reaction was terminated immediately upon completion.

#### 2.4.2. Bactericidal Efficacy of the Sequential Treatment of SAEW Followed by LTP Treatment

A concentration of available chlorine in SAEW and a corresponding treatment time were selected for the initial treatment of the bacterial suspension. The reaction was terminated immediately after this first step. Subsequently, the treated bacterial suspension was transferred to the LTP equipment for secondary treatment according to operational parameters.

#### 2.4.3. Bactericidal Efficacy of Combined Treatment of LTP and SAEW

The bacterial suspension was treated synchronously under the parameters of SAEW and LTP, and the treatment time was kept consistent with that used for LTP alone.

#### 2.4.4. Evaluation of Bactericidal Efficacy

With the untreated bacterial suspension as the control, the bacterial suspension from each aforementioned treatment group and their corresponding control groups were subjected to serial dilution. The diluted solutions were plated onto agar plates and incubated for 24 h. The bactericidal efficacy was assessed to identify the optimal treatment sequence.

### 2.5. Determination of Optimal Treatment Parameters of the Optimal Treatment Sequence

#### 2.5.1. Orthogonal Experimental Design

Single-factor experiments showed that LTP power, electrode spacing, SAEW ACC and treatment time, each had a significant effect on bactericidal efficacy (*p* < 0.05). Based on these results, these four factors, each at three levels, were selected for orthogonal optimization. To explore whether complete inactivation could be achieved at lower intensities by combining LTP and SAEW, the factor levels were set below the optimal conditions determined for the individual treatments. As shown in Table 1, an L_9_ (3^4^) orthogonal array was employed to design the experiments [30]. Using the logarithmic value of viable bacterial count as the evaluation metric, range analysis and analysis of variance (ANOVA) were performed to optimize the sterilization process parameters. The optimal treatment parameters for complete *L. monocytogenes* inactivation were identified based on the established optimal sequence of the treatment.

#### 2.5.2. Confocal Laser Scanning Microscopy Observation

After inactivation under the optimized conditions determined by orthogonal experiments, the *L. monocytogenes* suspension was collected and centrifuged at 3000× *g* for 5 min. The supernatant was discarded, and the pellet was washed twice with sterile phosphate-buffered saline (PBS) to remove residual treatment agents. The pellet was then resuspended in SYTO 9/PI staining solution, gently pipetted to mix, and incubated at room temperature in the dark for 15 min. Following staining, an equal volume of 40% glycerol in PBS was added and mixed gently. A small aliquot of the stained suspension was placed on a glass slide and covered with a coverslip.

Fluorescence images were acquired using a confocal laser scanning microscope (TCS SP5, Leica, Germany). SYTO 9 (green, live bacteria) and PI (red, dead bacteria) signals were collected with excitation/emission settings of 488 nm/500–550 nm and 561 nm/580–650 nm, respectively [31]. Sequential scanning mode was employed to capture images, allowing observation of bacterial morphology and the distribution of live and dead cells.

#### 2.5.3. VBNC Resuscitation Assay

The bacterial suspension treated under the optimal conditions (SAEW+LTP), the untreated bacterial suspension (positive control), and sterile saline (negative control) were separately inoculated into Brain Heart Infusion (BHI) broth (Beijing Land Bridge Technology Co., Ltd., Beijing, China). Enrichment was carried out at 37 °C with shaking at 160 rpm for 48 h. At 0, 6, 12, 18, 24, 30, 36, 42, and 48 h of enrichment, after thorough mixing, the OD_600_ value of the culture was measured using a SPECTROstar Nano microplate reader (BMG LABTECH, Ortenberg, Germany) by transferring 200 μL aliquots to a 96-well plate, with sterile BHI broth serving as the blank control. In parallel, at 0, 24, and 48 h of enrichment, 100 μL aliquots were taken and spread onto Lysogeny Broth (LB) agar (Beijing Land Bridge Technology Co., Ltd., Beijing, China), a non-selective medium, and PALCAM agar, a selective medium for *L. monocytogenes*. Each time point was tested in triplicate for each group. All plates were incubated at 37 °C for 24 h prior to colony observation [32,33].

### 2.6. Determination of Intracellular Component Leakage

#### 2.6.1. Determination of Protein Content

In this study, the Coomassie Brilliant Blue G-250 staining method was employed to determine protein content [34]. First, a 0.1 mg/mL bovine serum albumin (BSA) standard solution and Coomassie Brilliant Blue G-250 staining solution were prepared. Then, 0, 20, 40, 60, 80, and 100 μL of the BSA standard solution were sequentially added to a 96-well plate, and deionized water was added to make up the volume to 100 μL for standard curve preparation. The bacterial suspensions subjected to treatment and the untreated (control) bacteria suspension were collected, centrifuged at 10,000× *g* for 10 min, and the supernatants were obtained. A 100 μL aliquot of each supernatant from the control and treatment groups was added to the wells as unknown samples. Following the addition of 200 μL of Coomassie Brilliant Blue G-250 staining solution, the mixture was incubated for 2 min. The absorbance at 595 nm was recorded with a microplate reader, and protein concentration was calculated based on the standard curve to evaluate the extent of intracellular protein leakage.

#### 2.6.2. Determination of Nucleic Acid Content

The bacterial suspensions subjected to treatment and the untreated suspensions (served as the control group) were collected, centrifuged at 10,000× *g* for 10 min, and the supernatants were harvested. The ultraviolet spectrophotometry method was used to measure the absorbance of the treated supernatants at a wavelength of 260 nm. The leakage of intracellular nucleic acids was evaluated by comparing the nucleic acid content in the supernatant of the bacterial suspension between the treated and control groups. The concentration of nucleic acid was calculated according to the absorbance at 260 nm (A_260_).

(2) C = 50 × A_260_ × DF

where C is nucleic acid concentration (μg/mL), A_260_ is absorbance at 260 nm, DF is dilution factor, and 50 is the constant for double-stranded DNA (dsDNA).

### 2.7. Cell Morphology Observation

The scanning electron microscope (SEM; Model FEI-XI30) used in this study was purchased from Philips Electron Optics Co., Ltd. (Eindhoven, The Netherlands). Treated and untreated L. monocytogenes samples were collected and centrifuged at 8000× *g* at 4 °C for 5 min. After discarding the supernatant, the pellet was fixed overnight with 2.5% glutaraldehyde. The fixed cells were collected by centrifugation, washed twice with 0.1 mol/L phosphate buffer (PBS, pH 7.2–7.4), and then dehydrated in a graded ethanol series (30%, 50%, 70%, 80%, 90%, and 100%) for 15 min per concentration. Following drying, the samples were coated with gold. The morphology of the cells was observed using the SEM.

### 2.8. Statistical Analysis

All experiments were performed in triplicate, with three independent biological replicates and three technical replicates per independent experiment. Data are presented as mean ± standard deviation (SD). Statistical analysis was conducted using Duncan’s multiple range test after confirming the homogeneity of variances and normality of the data. Statistical significance was set at *p* < 0.05.

## 3. Results

### 3.1. Determination of Optimal Treatment Parameters

#### 3.1.1. Influence of Different Low Temperature Plasma Treatment Parameters on the Inactivation

**Figure 1:**
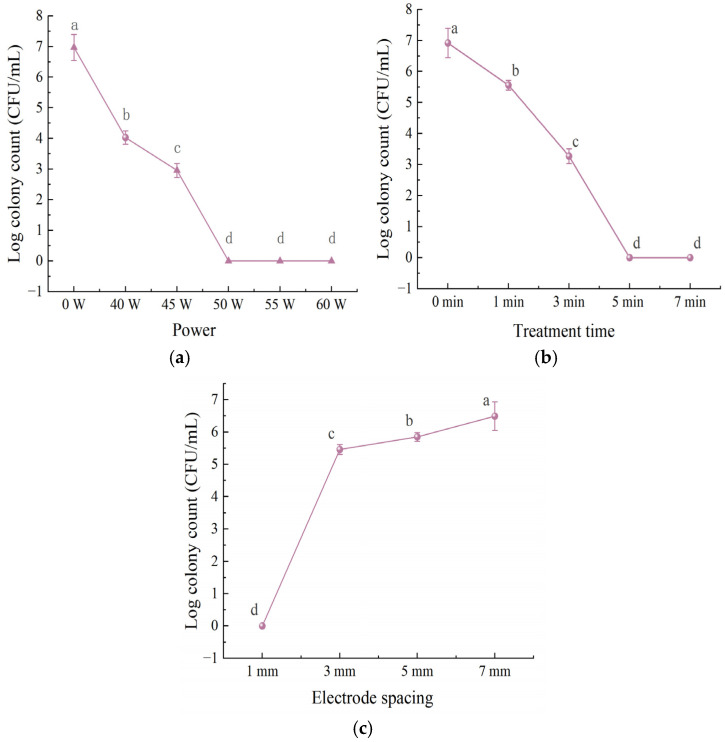
Bactericidal efficacy of LTP against *L. monocytogenes* under different powers (**a**), treatment times (**b**), and electrode spacing (**c**). Each experiment was independently replicated three times, and results are presented as mean values. Values with different letters differ significantly (*p* < 0.05).

In Figure 1, the bactericidal efficacy of LTP against *L. monocytogenes* increased significantly with increasing applied power from 40 to 60 W. Compared with the control group (6.97 log CFU/mL), complete inactivation was achieved at 50 W, with the viable count falling below the detection limit (<1.0 log CFU/mL).

Treatment time also significantly affected the bactericidal efficacy of LTP. Compared with the control group (6.92 log CFU/mL), the viable count decreased significantly after 1 min of exposure (*p* < 0.05). Complete inactivation was achieved after 3 min, and the viable count remained below the detection limit at 5 and 7 min. Complete inactivation was achieved at 1 mm spacing, the bacterial count rose to 5.46 log CFU/mL at 3 mm, and further increased to 6.49 log CFU/mL at 7 mm, showing a severe decline in antimicrobial performance.

#### 3.1.2. Influence of Different Slightly Acidic Electrolyzed Water Treatment Parameters on the Inactivation

**Figure 2:**
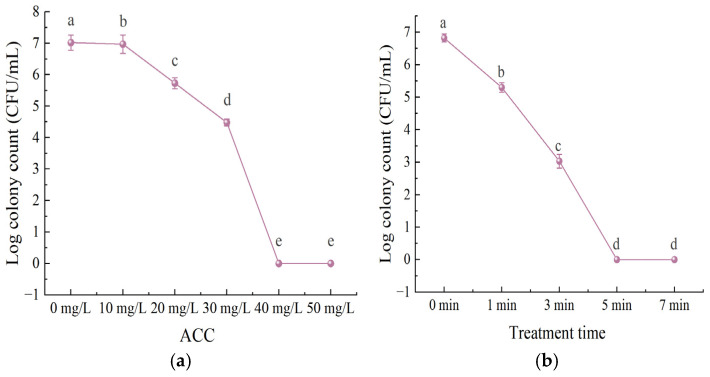
Bactericidal efficacy of SAEW against *Listeria monocytogenes* under different available chlorine concentrations (**a**) and treatment times (**b**). Each experiment was independently replicated three times, and results are presented as mean values. Values with different letters differ significantly (*p* < 0.05).

In Figure 2, with the control group (*L. monocytogenes* count: 7.02 log CFU/mL) as the baseline, the bactericidal activity of SAEW against the target bacteria increased significantly with the gradient elevation of ACC in the range of 10 mg/L to 50 mg/L, and the viable count fell below the detection limit when ACC reached 40 mg/L or above.

With the control group as the reference (colony count of *L. monocytogenes* at 6.82 log CFU/mL), the bactericidal activity of SAEW against the target bacteria showed a continuous and significant increasing trend with the extension of treatment time, and after 5 min of treatment, the viable count fell below the detection limit.

#### 3.1.3. Influence of Different Treatment Sequences in Combined Treatment Methods on the Inactivation

**Figure 3:**
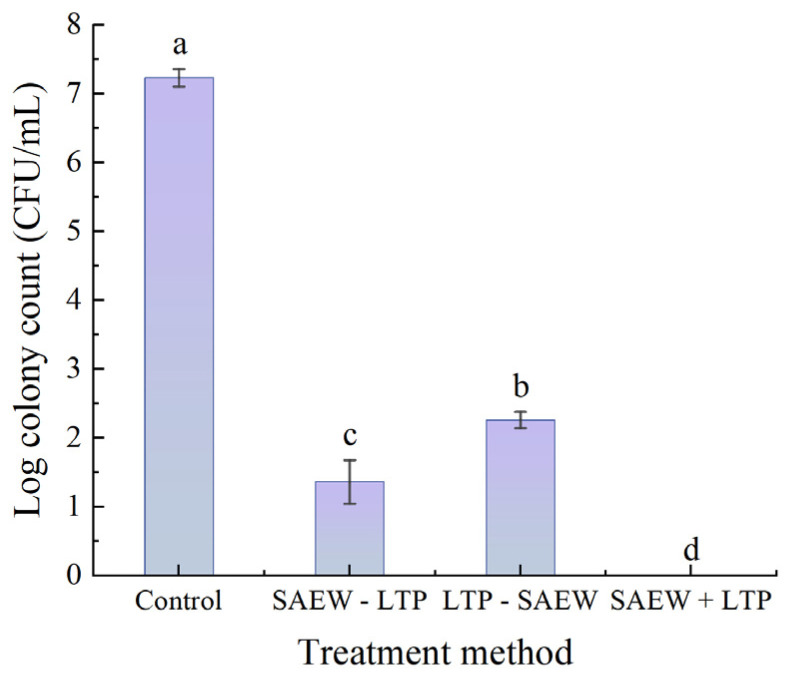
Bactericidal efficacy of combined sterilization treatments with different sequences against *Listeria monocytogenes*. Each experiment was independently replicated three times, and results are presented as mean values. Values with different letters differ significantly (*p* < 0.05). Control: blank control group; SAEW-LTP: treatment group of SAEW followed by LTP; LTP-SAEW: treatment group of LTP followed by SAEW; SAEW+LTP: combined treatment group with SAEW and LTP.

Combined treatment experiments were performed with LTP at 45 W, 3 min treatment time and 1 mm electrode spacing and SAEW at 30 mg/L ACC with a 3 min treatment time. In Figure 3, statistical analysis showed that all three combined treatment methods had significant bactericidal activity against *L. monocytogenes*, with notable differences in efficacy based on treatment sequence. Compared with the control group (bacterial count: 7.23 log CFU/mL), the SAEW+LTP treatment exhibited the highest bactericidal efficacy, with the viable count falling below the detection limit. The SAEW-LTP treatment ranked second, reducing the bacterial count by 5.87 log, while the LTP-SAEW treatment resulted in a 4.97 log reduction. All three combined treatment sequences showed superior bactericidal effects compared to single LTP or SAEW treatment.

#### 3.1.4. Determination of Optimal Parameters

Four key influencing factors (LTP power, treatment time, SAEW ACC, electrode spacing) with three levels each were selected, and an L_9_ (3^4^) orthogonal experiment was designed with the logarithmic value of viable bacterial count as the evaluation metric. Range analysis of the results (Table 2) showed the order of factor influence by range value magnitude as R4 > R1 > R3 > R2, i.e., electrode spacing > power > ACC > treatment time. The optimal parameter combination was identified as A_3_B_2_C_3_D_1_, corresponding to an LTP power of 45 W, a treatment time of 2 min, an SAEW ACC of 30 mg/L, and an electrode spacing of 1 mm. Validation experiments (Table 3) confirmed that this optimal combination achieved complete inactivation of *L. monocytogenes*, thus verifying the feasibility of the established protocol.

### 3.2. Validation of Inactivation by Confocal Laser Scanning Microscope (CLSM)

**Figure 4:**
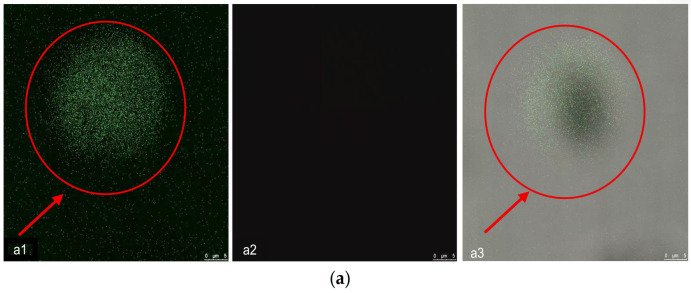

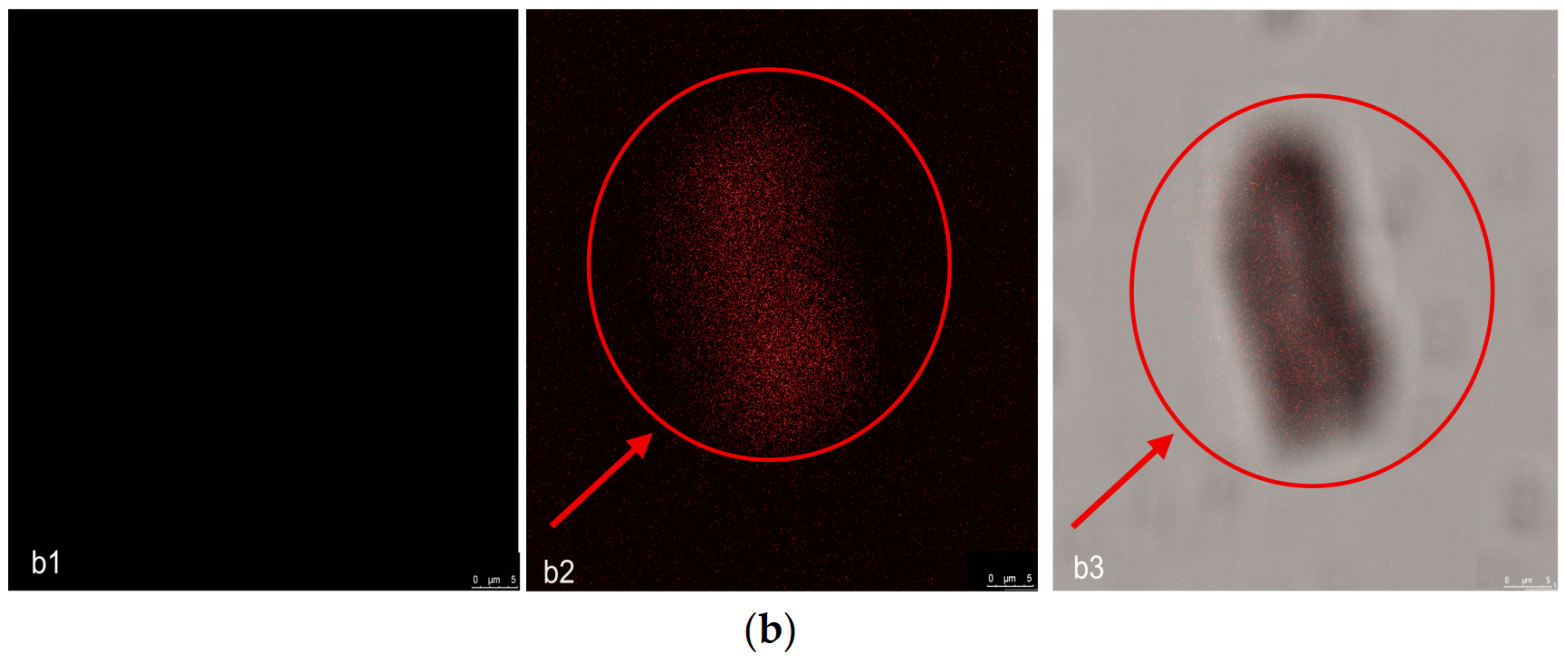
CLSM observation of *L. monocytogenes* following combined treatment with SAEW and LTP. For each sample, three random fields of view were examined. (**a**) Untreated control cells: (**a1**) SYTO 9 (green) channel; (**a2**) PI (red) channel; (**a3**) merged image. (**b**) Cells treated with SAEW+LTP: (**b1**) SYTO 9 (green) channel; (**b2**) PI (red) channel; (**b3**) merged image.

To further verify bacterial viability under the optimized conditions, CLSM with SYTO 9/PI staining was performed. In the control group, bright green fluorescence was observed in the green channel with no signal in the red channel, and the merged image appeared green (Figure 4a). In contrast, the SAEW+LTP treatment group exhibited no green fluorescence, whereas strong red fluorescence was detected in the red channel, with the merged image displaying a red signal (Figure 4b). These results indicate cell death and membrane damage, consistent with the plate count results presented in Section 3.1.3.

### 3.3. VBNC Resuscitation Assay

**Figure 5:**
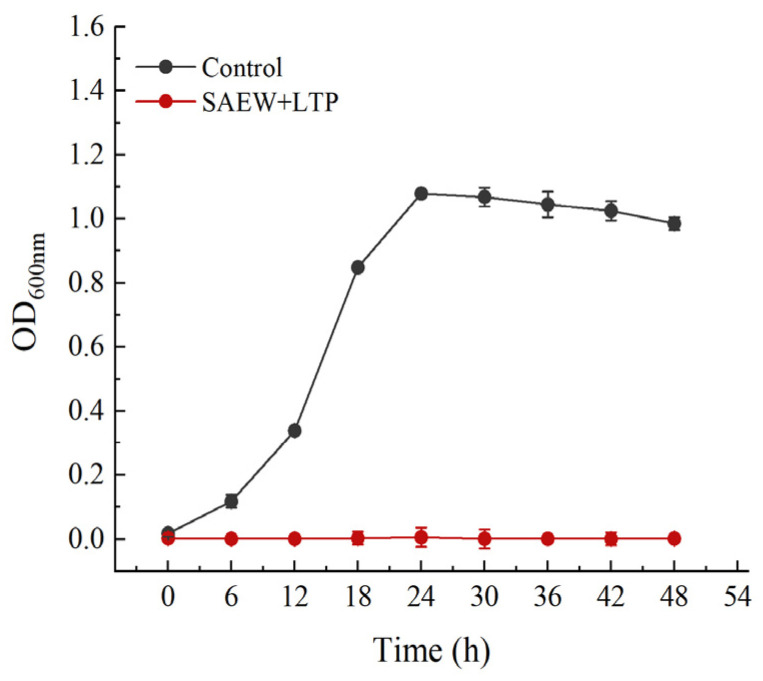
Growth curves of *L. monocytogenes* in BHI broth over 48 h. The untreated bacterial suspension showed a typical growth curve, while the SAEW+LTP-treated suspension showed no detectable increase in OD_600_ throughout the 48 h enrichment. Data are presented as mean ± SD (*n* = 3).

**Figure 6:**
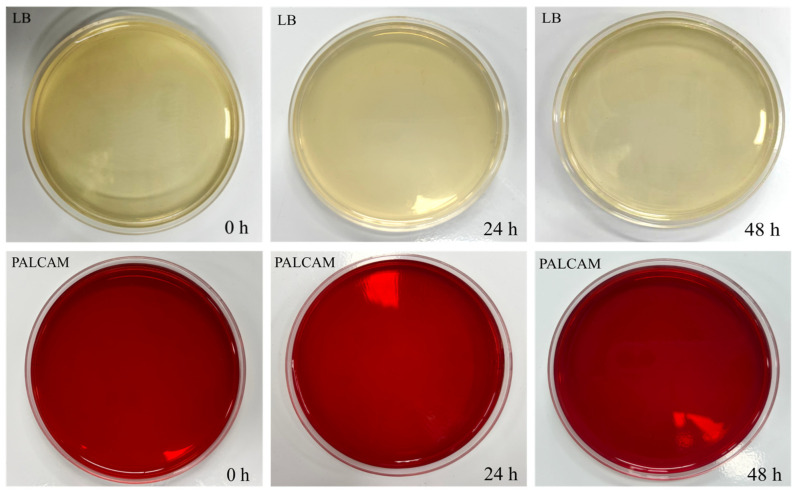
Colony formation of *L. monocytogenes* on LB and PALCAM agar after SAEW+LTP treatment. The bacterial suspension treated under optimal conditions (SAEW+LTP) was plated undiluted (100 μL per plate) onto LB agar and PALCAM agar (selective for *L. monocytogenes*) at 0, 24, and 48 h of enrichment.

As shown in Figure 5, the untreated bacterial suspension showed a typical growth curve, increasing rapidly then gradually stabilizing, while the OD_600_ value of the SAEW+LTP-treated suspension remained stable throughout the 48 h enrichment, indicating no detectable increase in turbidity. In parallel, colony formation was examined at 0, 24, and 48 h of enrichment (Figure 6). No colonies were observed for the treated group on either LB aga or PALCAM agar (selective for *L. monocytogenes*). The positive control showed normal growth on both media at 0, 24, and 48 h, with colony counts increasing over time, while the negative control showed no growth at any time point (Supplementary Figures S1 and S2).

### 3.4. Analysis of Cellular Damage

#### 3.4.1. Influence of Treatment on Bacterial Protein and Nucleic Acid Leakage

**Figure 7:**
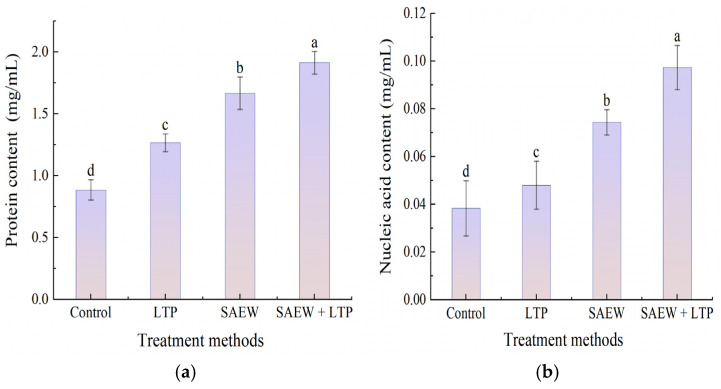
Protein content (**a**) and nucleic acid content (**b**) with different treatments. Each experiment was independently replicated three times, and results are presented as mean values. Values with different letters differ significantly (*p* < 0.05). Treatment methods: Control = blank control group; LTP = LTP-only treatment group; SAEW = SAEW-only treatment group; SAEW+LTP = combined treatment group with SAEW and LTP.

The results of protein and nucleic acid leakage measurements are shown in Figure 7 (a: protein, b: nucleic acid). Absorbance at OD_260_ nm and OD_280_ nm reflects changes in cell membrane permeability, both individual and combined treatments significantly induced the leakage of intracellular proteins and nucleic acids in *L. monocytogenes*. The leakage level in all treatment groups was significantly higher than that in the control group, following the order: SAEW+LTP > SAEW > LTP. The protein and nucleic acid leakage in the SAEW+LTP treatment group reached 1.92 mg/mL and 0.097 mg/mL, respectively, with increases of 123.3% and 155.3% compared to the control. The SAEW treatment group showed moderate leakage effects, while the LTP treatment group had relatively lower increases of 47.7% and 26.3%. The combined treatment induced significantly greater intracellular leakage than the sum of the individual LTP and SAEW treatments.

#### 3.4.2. Observation of Bacterial Cell Morphology

**Figure 8:**
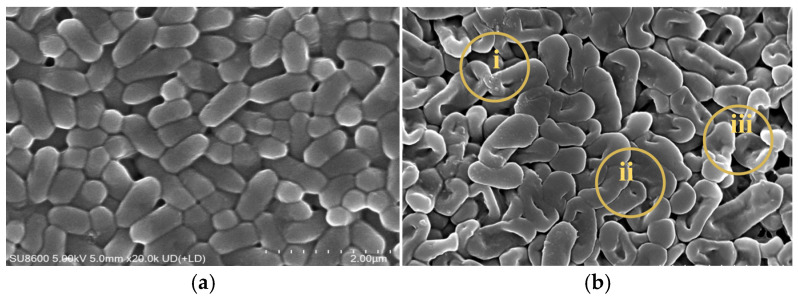
Scanning electron microscopy images of *Listeria monocytogenes* before (**a**) and after (**b**) treatment. Regions i, ii, and iii highlight the main morphological changes.

Scanning electron microscopy (SEM) observations revealed multiple morphological changes in *L. monocytogenes* treated with the combined method compared to the untreated group. As can be seen from Figure 8, at region “i”, the original regular short rod shape disappeared, and the cells showed irregular forms such as indentations and twists. At region “ii”, the cell surface became rough instead of smooth. At region “iii”, cell boundaries were blurred and some cells showed rupture or perforation.

## 4. Discussion

The trend that the bactericidal efficacy of LTP was enhanced with increasing power was in agreement with the findings of Zhang et al. [35], confirming the key role of power and the rationality of the optimal power parameter of 50 W in this study. The significant regulatory effect of treatment time on bactericidal efficacy was echoed by the findings of Du et al. [36], who reported that prolonging LTP treatment time could improve bactericidal efficacy. This strongly supports the conclusion that, in the present study, the viable count fell below the detection limit after 5 min of treatment. Moreover, the observed significant negative correlation between electrode spacing and LTP bactericidal efficiency was consistent with the results of Dahle et al. [37], who found that widening the electrode spacing led to a continuous decline in bactericidal efficiency, further verifying its important regulatory role in the bactericidal effect of LTP.

For SAEW, the bactericidal activity against *L. monocytogenes* was significantly enhanced with the gradual increase of ACC, which was consistent with the findings of Duan et al. [38]. The viable count fell below the detection limit when ACC reached 40 mg/L or above, further confirming the influence of ACC on the bactericidal efficacy of SAEW. Meanwhile, the bactericidal activity of SAEW against the target bacteria showed a continuous and significant increasing trend with the extension of treatment time, which was in agreement with the trend reported by Wu et al. [39]. The viable count fell below the detection limit after 5 min of treatment, indicating that treatment time also plays an important role in the bactericidal efficacy of SAEW.

The bactericidal efficiency of different combined treatments varied significantly, but all were superior to those of single treatments. Among the three treatment sequences, the SAEW+LTP combined treatment exhibited the most significant bactericidal effect against *L. monocytogenes*, with viable counts falling below the detection limit. Furthermore, its bactericidal efficacy exceeded the sum of the individual effects of LTP and SAEW alone. This phenomenon may be related to the cell wall characteristics of Gram-positive bacteria, their peptidoglycan layer lacks an outer membrane barrier, allowing hypochlorous acid in SAEW to easily penetrate the cell wall and further damage intracellular proteins. LTP generates reactive oxygen species (ROS) and reactive nitrogen species (RNS), which can further oxidize nucleic acids [40], while the precise mechanism remains to be elucidated. Orthogonal experimental results revealed that electrode spacing was the key factor influencing the combined bactericidal efficacy. A slight increase in spacing sharply reduced the concentration of plasma active substances and thus the bactericidal efficacy. The optimal parameters (LTP 45 W, 1 mm electrode spacing, 2 min; SAEW 30 mg/L ACC) resulted in viable counts below the detection limit, with both energy input and ACC being lower than those required for individual treatments.

CLSM observation revealed that the optimized treatment compromised the cell membrane integrity of *L. monocytogenes*, leading to loss of viability, providing direct evidence for the bactericidal efficacy of the combined approach.

The VBNC state represents a reversible dormant state that bacteria enter under unfavorable conditions, from which they can resume growth when conditions become favorable. As a foodborne pathogen, *L. monocytogenes* poses a potential threat to food safety due to its ability to enter the VBNC state [41]. To determine whether the combined SAEW+LTP treatment induced a VBNC state, a resuscitation assay was performed in this study. The results showed that after 48 h of enrichment in BHI broth, the treated bacterial suspension exhibited no significant change in OD_600_ and formed no colonies on either non-selective LB agar or selective PALCAM agar, indicating no detectable resuscitated growth. Together with the CLSM observations of irreversible damage to cell membrane integrity, these results confirm that the combined SAEW+LTP treatment leads to complete inactivation of *L. monocytogenes*, eliminating the risk of subsequent resuscitation.

Collectively, these findings demonstrate that the combination of LTP and SAEW effectively inactivates *L. monocytogenes*. Under the optimized conditions, complete inactivation was achieved with energy input and chemical usage significantly lower than those required for individual treatments. This highlights a key advantage of the combined approach and aligns with the demand for green and efficient food sterilization in industrial applications.

Regarding cellular injury, the findings on protein and nucleic acid leakage in this study are consistent with the bactericidal mechanism reported by Gao et al. [42]. This further confirms that the antimicrobial effect of the SAEW+LTP combined treatment is not a simple additive effect, but an enhancement via complementary mechanisms of action [43]. Overall, the combined treatment can effectively inactivate *L*. *monocytogenes* by damaging its cell membrane structure, resulting in massive leakage of key intracellular metabolites and complete loss of physiological function.

According to the SEM observations, the irregular cell morphology in region “i” may be attributed to the attack of active substances in LTP and strong oxidizing substances in SAEW, which destroy the peptidoglycan and other structural support components [27]. The rough cell surface in region “ii” resulted from surface damage caused by high-energy particles in LTP and oxidation of surface proteins and lipids by SAEW, further impairing cellular functions such as substance transport and signal recognition [44]. In region “iii”, the combined effect of LTP and SAEW severely disrupted the integrity of the cell wall and membrane, leading to intracellular material leakage, homeostatic imbalance, and eventual cell death. Furthermore, damage to cell morphology and structure inhibited physiological functions and blocked key processes including energy metabolism and DNA replication, ultimately resulting in the inactivation of *L*. *monocytogenes*.

This study demonstrated that the combined treatment of LTP and SAEW exerted a potent bactericidal effect against *L. monocytogenes* ATCC 19114, with energy input substantially lower than that required for individual treatments. The equipment features a simple configuration, and SAEW can be generated on-site by electrolysis, making the combined approach easy to operate and cost-effective. Future research should focus on elucidating the molecular mechanisms underlying this combined approach, integrating transcriptomic and proteomic analyses to unravel its regulatory networks. Further validation in real food matrices is warranted to assess the impact of food components on bactericidal efficacy and food quality. Additionally, extending the investigation to include *L. monocytogenes* strains of different serotypes and origins would help clarify the broad-spectrum applicability of this technology, thereby providing critical support for its practical implementation in the food processing industry.

## 5. Conclusions

The combined treatment of LTP and SAEW effectively inactivated *Listeria monocytogenes*. Under the optimal parameters (LTP: 45 W, 1 mm for 2 min; SAEW: 30 mg/L), loss of culturability and cell death were achieved, as confirmed by plate count and CLSM observation. A resuscitation assay further ruled out the VBNC state. Analysis of cellular damage revealed that the combined treatment compromised cell membrane integrity, leading to leakage of intracellular contents, and caused marked morphological disruption, as observed by SEM. Collectively, these findings indicate that the combined approach disrupts normal physiological functions of the bacteria, thereby achieving efficient bactericidal activity.

## Figures and Tables

**Table 1:** Orthogonal experimental design table.

Level	A	B	C	D
	Power (W)	Treatment Time (min)	ACC (mg/L)	Electrode Spacing (mm)
1	35	1	20	1
2	40	2	25	2
3	45	3	30	3

**Table 2:** Results and analysis of orthogonal experiments.

No.	A	B	C	D	Log Value of Viable Bacteria
1	1	1	1	1	2.19
2	1	2	2	2	3.93
3	1	3	3	3	4.06
4	2	1	2	3	3.31
5	2	2	3	1	0.15
6	2	3	1	2	2.96
7	3	1	3	2	2.42
8	3	2	1	3	3.03
9	3	3	2	1	0.30
K_1_	10.17	7.92	8.18	2.64	
K_2_	6.43	7.10	7.54	9.31	
K_3_	5.74	7.32	6.63	10.40	
k_1_	3.39	2.64	2.73	0.88	
k_2_	2.14	2.37	2.51	3.10	
k_3_	1.91	2.44	2.21	3.47	
Range (R)	1.48	0.27	0.52	2.59	
Optimal scheme	A3	B2	C3	D1	

A, low temperature plasma power (W): level 1 = 35, level 2 = 40, level 3 = 45; B, treatment time (min): level 1 = 1, level 2 = 2, level 3 = 3; C, available chlorine concentration (mg/L): level 1 = 20, level 2 = 25, level 3 = 30; D, electrode spacing (mm): level 1 = 1, level 2 = 2, level 3 = 3. The viable bacterial count is expressed as log CFU/mL, and data represent the mean of three independent biological replicates. K_1_, K_2_, and K_3_ represent the sum of the results at levels 1, 2, and 3, respectively; k_1_, k_2_, and k_3_ represent the corresponding means; R represents the range (Kmax−Kmin).

**Table 3:** Verification of the optimal scheme.

No.	Power	Treatment Time	ACC	Electrode Spacing	Log Value of Viable Bacteria
Control	-	-	-	-	6.87 ± 0.07 ^a^
SAEW+LTP	45 W	2 min	30 mg/L	1 mm	<1.0 ^b^

Data are presented as mean ± standard deviation (*n* = 3). Superscript letters ^a^ and ^b^ in the same column indicate significant differences (*p* < 0.05). The detection limit was 1.0 log CFU/mL (based on a plating volume of 100 μL).

## Data Availability

The original contributions presented in this study are included in the article/Supplementary Materials. Further inquiries can be directed to the corresponding authors.

## Supplementary Materials

The following supporting information can be downloaded at: https://www.mdpi.com/article/10.3390/foods15091458/s1, Figure S1: Positive control of the VBNC resuscitation assay. Untreated L. monocytogenes suspension was plated after 10^6^-fold dilution (100 μL) onto LB agar and PALCAM agar (selective for *L. monocytogenes*) at 0, 24, and 48 h of enrichment. The positive control showed normal growth on both media at all time points, with colony counts increasing over time; Figure S2: Negative control of the VBNC resuscitation assay. Sterile saline was plated undiluted (100 μL per plate) onto LB agar and PALCAM agar (selective for *L. monocytogenes*) at 0, 24, and 48 h of enrichment. No colonies were observed on either medium at any time point.

## Abbreviations

The following abbreviations are used in this manuscript:

LTP	Low Temperature Plasma
SAEW	Slightly Acidic Electrolyzed Water
ACC	Available Chlorine Concentration
PBS	Phosphate Buffer
SEM	Scanning Electron Microscopy
OD	Optical Density
BSA	Bovine Serum Albumin
TSB-YE	Tryptone Soy Broth with Yeast Extract
ROS	Reactive Oxygen Species
RNS	Reactive Nitrogen Species
CFU	Colony Forming Unit
CLSM	Confocal Laser Scanning Microscopy
BHI	Brain Heart Infusion
LB	Lysogeny Broth

## References

[1] Li H.Q., Guo Y.C., Liu Z.T., Song J., Zhou L., Yang X.R., Jia H.Y., Liu J.K., Li W.W., Han H.H. (2024). Analysis of foodborne disease outbreaks in China’s Mainland in 2022. Chin. J. Food Hyg..

[2] Xue J., Sun F.L., Cheng M., Sun C. (2025). Surveillance results of foodborne disease in a sentinel hospital in Weifang, Shandong from 2019 to 2023. Mod. Dis. Control. Prev..

[3] Zhang P. (2025). The easily overlooked “cold killer”—*Listeria monocytogenes*. Fam. Med..

[4] Gong C.B., Dong F.G., Wang Z.X., Sun Y.L. (2013). Risk assessment and investigation of *Listeria monocytogenes* contamination of meat and meat products at retail in Yantai. Food Sci. Technol..

[5] Shen S.W. (2020). Overview of food sterilization technologies. China Food Saf. Mag..

[6] Wan J.N., Zhao Y.T., Li H.H., Zhao Y.H., Wang C.F., Jiang H., Zhang X.N. (2025). A comparative analysis of thermal and non-thermal sterilization processing: Effects on the digestive characteristics and antioxidant bioactivity of donkey whey protein in a simulated gastrointestinal model for infants. Food Biosci..

[7] Wang N.J. (2026). Application of Food Preservation Technology in Prepared Dishes. Food Ind..

[8] Wang X.D., Kong Y.Z., Zhang Y.L., Tsogtbayar A., Burenjargal M., Wu H.X., Dong A.L.D.E.T. (2022). Mechanism of sterilization technology and its application in food field. China Brew..

[9] Xue L. (2023). Ultrasonic Technology and Its Application in Food Industry. Food Ind..

[10] Zheng R.W., Xiao H., Liu S.J., Yang Q., Wang J.Y., Ye Y. (2017). Effect of High Voltage Pulsed Electric Field on the Quality of Liquid Food. Food Res. Dev..

[11] Xie D.H., Yang M.H., Zhu M.G., Zhang L.Y., Huang G.H., You Y.H., Zhou W.J. (2025). Active packaging film with synergistic effect of UV-shielding and photodynamic sterilization for strawberry preservation. Food Packag. Shelf Life.

[12] Ma X.L., Yu X.G., Li X.W., Hu G.Y. (2024). Research Progress on Prepared Dishes Processing of Edible Mushroom. Food Sci. Technol..

[13] Nakthong N., Tuntithavornwat S., Eshtiaghi M.N. (2025). Impact of combined nano natural antimicrobials and pulsed electric field on pineapple juice preservation. Appl. Food Res..

[14] Ma Y.F., Ma Y.Q., Gong W.J., Chi L., Xiang Q.S. (2024). Combined Antimicrobial Effect of Plasma-Activated Water and Mild Heat Against Penicillium expansum Spores. LWT.

[15] Yang M., Chao H.J., Hou Z.H., Wang L.L., Xu W.Z., Zhao X. (2025). Antimicrobial Activity of Octyl Gallate Nanoemulsion Combined with Photodynamic Technology and Its Effect on Food Preservation. Int. J. Food Microbiol..

[16] Chavan P., Prendeville J., Hamid, Jaiswal S., Jaiswal A.K. (2024). Chapter 12—Cold plasma treatment in food packaging: Effects on material properties, sterilization, and safety considerations. Food Packag. Preserv..

[17] Ren H.R., Quan Y., Liu S.K., Hao J.X. (2025). Effectiveness of ultrasound (US) and slightly acidic electrolyzed water (SAEW) treatments for removing *Listeria monocytogenes* biofilms. Ultrason. Sonochemistry.

[18] Hu R.H., Tan B. (2023). Research Progress on Application of New Food Sterilization Technology. China Food Saf. Mag..

[19] Vedaei S., Dara A. (2025). Surveying the utilization of cold plasma and plasma-activated water on food pigments, bioactive compounds, enzymes, vitamins, fatty acid, and essential oils: Considerations, mechanisms, and future trends. Food Chem. X.

[20] Chen L., Fan M.Q., Li Y.J., Zheng X.C., Hao J.X. (2024). Application of Low Temperature Plasma Technology in the Food Industry: A Review. Food Res. Dev..

[21] Du Y.L., Tian Q., Li G.J., Yi J.J., Hu X.S., Jiang Y.L. (2024). Advanced application of slightly acidic electrolyzed water for fresh-cut fruits and vegetables preservation. Food Res. Int..

[22] Zhang Z.L. (2023). Study on Application of Acidic Electrolyzed Water in Food Industry. China Clean. Ind..

[23] Kong T.Y., Zhao X.D., Li G., Ma C., Zhang Q. (2026). Application Progress of Low Temperature Plasma Technology in Fruit and Vegetable Preservation. Food Sci. Technol..

[24] Lv Y.D., Zhao J.F., Jiang C.S., Liu Y., Yang F. (2025). Research progress on the application of slightly acicic electrolyzed water in postharvest vegetable preservation. China Cucurbits Veg..

[25] Lu S.Y., Hei J.X., Huang X.Q., Song W.X., Wei Z.W., Hai D., Song L.J., Shen Y. (2026). A Systematic Review of Ultrasound, Light and Their Synergistic Technologies in Food Sterilization. Sci. Technol. Food Ind..

[26] Barrales Astorga J., Hadinoto K., Cullen P., Prescott S., Trujillo F.J. (2022). Effect of plasma activated water on the nutritional composition, storage quality and microbial safety of beef. LWT.

[27] He X., Ren K., Xu C., Zhang Y.T., Zhou H.S., Ling J., Li P.X., Cheng S.C., Hu H.L. (2025). Inhibitory Effect of Slightly Acidic Electrolyzed Water on Spoilage Bacteria in Fresh-Cut Chicory. Food Sci..

[28] Miao J. (2024). Study on the Determination of Dissolved Oxygen in Water by Iodometric Method. Shanxi Chem. Ind..

[29] Li S.Y., He J.S., Ye S.X., Dong W.M., Gao Q. (2025). Comparison of the bactericidal effects of different parallel techniques on *Escherichia coli* on the surface of Panax notoginseng fresh slices. J. Food Saf. Qual..

[30] Liu L.H., Wang L.P., He H.N., Cao Y.L. (2022). Optimum of Zinc Oxide Recycled Process from Waste Zinc Oxide Catalyst by Orthogonal Experiment. Technol. Dev. Chem. Ind..

[31] Wang P., Tian L., Yang S.Q., Liu J.Q. (2026). Study on mechanism of thymol’s antibacterial action against *Listeria monocytogenes*. J. Shaanxi Univ. Sci. Technol..

[32] Luo K.Y., Hu X.Q., Li Y.Z., Guo M.X., Liu X., Zhang Y.Y., Zhuo W.W., Yang B.W., Wang X., Shi C. (2024). Revealing the mechanism of citral induced entry of Vibrio vulnificus into viable but non-culturable (VBNC) state based on transcriptomics. Int. J. Food Microbiol..

[33] Zhang Y.J., Xu R.T., Zhu X.N., Yang Z.Y., Bian Y.H., He X., Tang Y.L. (2025). UV-Induced VBNC state formation and resuscitation in *E. coli*: ATP-based metabolic activation and biofilm-mediated recovery. Water Res..

[34] Wan D.N. (2024). Study on Determination of Protein Content in Milk Powder by Coomassie Brilliant Blue Method. China Food Ind..

[35] Zhang G.T., Zhang D.J., Li J., Wang H.J., Jin L.D., Guan Y.H., Xu M.L. (2022). Advances in the Application of Cold Plasma Technology in Food Sterilization. Sci. Technol. Food Ind..

[36] Du W.H., Huang S.H., Li H., Yang C.J., Xu Y. (2015). In vitro inhibition of low-temperature plasma on *Candida albicans*. Acta Univ. Med. Anhui.

[37] Dahle S., Žigon J., Fink R. (2024). Cold Plasma for Sustainable Control of Hygienically Relevant Biofilms: The Interaction of Plasma Distance and Exposure Time. Int. J. Environ. Health Res..

[38] Duan H.Y., Wang J.Q., Shen J., Zhang W., Sun H.H., Zhang L.B., Ban H.Q. (2021). Microbial killing efficacy of slightly acidic electrolyzed water in laboratory. Chin. J. Infect. Control..

[39] Wu C.H., Kaneyasu Y., Yano K., Shigeishi H., Kitasaki H., Maehara T., Niitani Y., Takemoto T., Mine Y., Nguyen-Tra Le M. (2024). Anti-fungal Effects of Slightly Acidic Electrolyzed Water on Candida Species. J. Oral Biosci..

[40] Hao Y., Xu G.M., Shi X.M., Zhang G.J. (2024). Research Progress on Application of Low Temperature Plasma Disinfection and Sterilization Technology. Chin. J. Disinfect..

[41] Wang J.T., Li Z., Yang J.L., Dong Q.L. (2026). Resuscitation of viable but nonculturable foodborne pathogenic bacteria: Influencing factors, mechanisms, and physiological characteristics. Food Ferment. Ind..

[42] Gao C., Zhu J.H., Xie L.L., Kong Y.J., Cai X.L., Zhang C.L., Shan Z.G., Shi C. (2026). Synergistic Bactericidal Effect and Mechanism of Ultrasound Combinedwith Carvacrol Nanoemulsion on Salmonella Typhimurium and its Application to Cabbage. Mod. Food Sci. Technol..

[43] Chen Y., Wu Y.H., Zhou J.W., Liu D.H., Lv R.L. (2025). Bactericidal Effect and Mechanism of Ultrasound Combined with Ultraviolet Treatment against *Escherichia coli* O157:H7. Food Sci..

[44] Xuan X.T., Zhang Z.Y., Shang H.T., Sheng Z.L., Cui Y., Lin X.D., Chen S.Q., Zhu L. (2025). Microbial Diversity and Antibacterial Mechanism of Slightly Acidic Electrolyzed Water against Pseudomonas fluorescens in Razor Clam during Storage. Food Res. Int..

