# Supplementary material for: Bactericidal Effect of Low Temperature Plasma Combined with Slightly Acidic Electrolyzed Water Against Listeria monocytogenes
Source: Foods. 2026 Apr 22;15(9):1458. doi: 10.3390/foods15091458 (PMC13163760; doi:10.3390/foods15091458)
Supplement: Supplementary file 1 [file foods-15-01458-s001.zip › foods-4207208-supplementary.pdf]

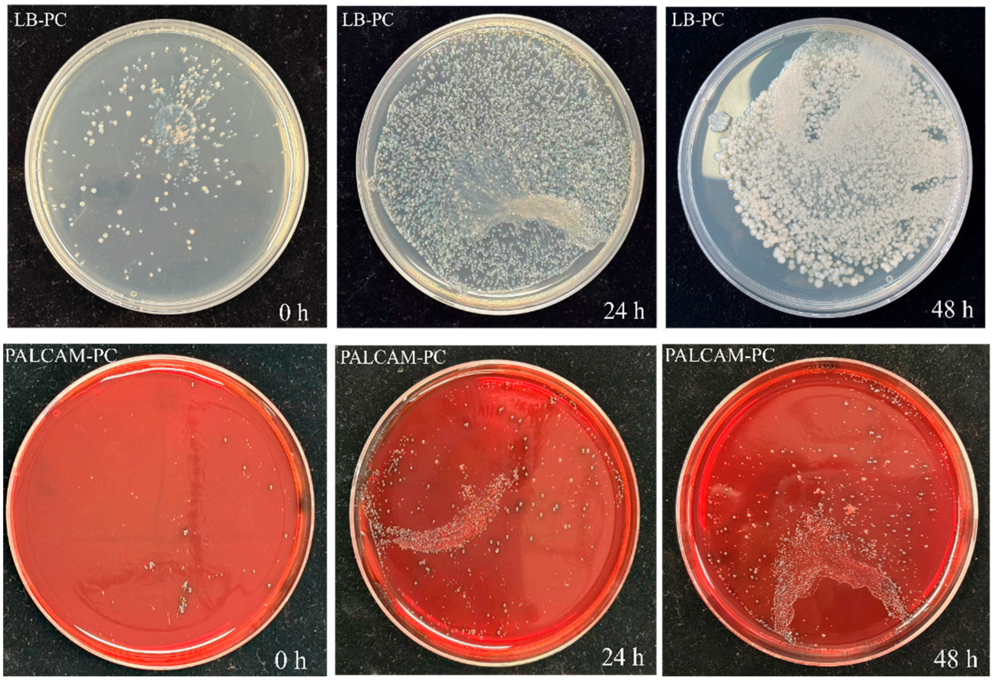

Figure **S1**. Positive control of the VBNC resuscitation assay. Untreated *L. monocytogenes* suspension was plated after 10^6-fold dilution (100 μL) onto LB agar and PALCAM agar (selective for *L. monocytogenes*) at 0, 24, and 48 h of enrichment. The positive control showed normal growth on both media at all time points, with colony counts increasing over time.

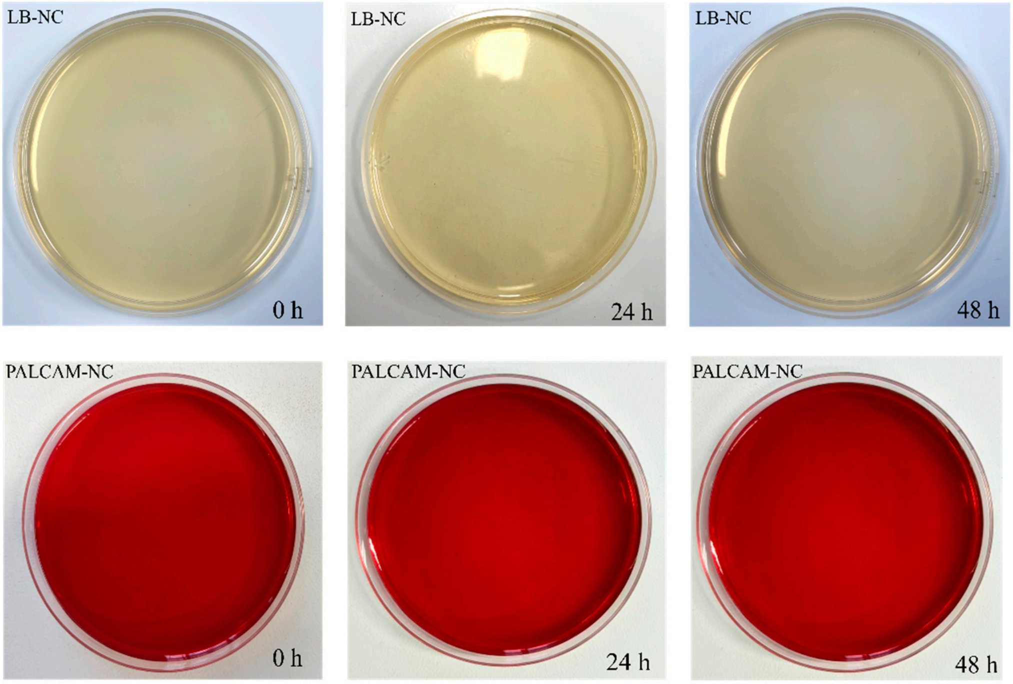

Figure **S2**. Negative control of the VBNC resuscitation assay. Sterile saline was plated undiluted (100 μL per plate) onto LB agar and PALCAM agar (selective for *L. monocytogenes*) at 0, 24, and 48 h of enrichment. No colonies were observed on either medium at any time point.
